# O Estudo ACCEPT e o Manejo da Doença Isquêmica Miocárdica no Brasil: Desafios e Oportunidades em um País Continental

**DOI:** 10.36660/abc.20240604

**Published:** 2024-11-13

**Authors:** Henrique Tria Bianco

**Affiliations:** 1 Universidade Federal de São Paulo Escola Paulista de Medicina São Paulo SP Brasil Escola Paulista de Medicina – Universidade Federal de São Paulo, São Paulo, SP – Brasil

**Keywords:** Infarto do Miocárdio, Síndrome Coronariana Aguda, Registro Médico Coordenado

A doença isquêmica miocárdica, particularmente em sua manifestação aguda como síndrome coronariana aguda (SCA), permanece como um dos principais desafios de saúde pública no Brasil e no mundo.^1,2^ Neste contexto, o estudo ACCEPT (*Acute Coronary Syndrome Registry*) emerge como uma fonte crucial de informações epidemiológicas e clínicas sobre o manejo das SCA no cenário brasileiro, com o objetivo de avaliar as terapias baseadas em evidência, a ocorrência de desfechos, uso de reperfusão e preditores para não receber reperfusão nos pacientes com SCACEST em um registro nacional multicêntrico.^3,4^

O Brasil, com suas dimensões continentais e marcantes disparidades regionais, apresenta um desafio único na implementação de estratégias uniformes para o tratamento das SCA. O estudo ACCEPT não apenas fornece dados valiosos sobre a prevalência e os tipos de SCA encontrados no país, mas também lança luz sobre as diferentes abordagens terapêuticas adotadas em diversas regiões.^5^ A revascularização miocárdica é um pilar fundamental no tratamento das SCA, especialmente no infarto agudo do miocárdio com supradesnivelamento do Segmento-ST (IAMCSST).^6^ O ACCEPT demonstra que, embora a ICPp (Intervenção Coronária Percutânea Primária), primária seja considerada o padrão-ouro para o IAMCSST, sua disponibilidade e aplicação variam significativamente entre as regiões brasileiras. Em áreas metropolitanas e centros de referência, a ICP primária é frequentemente a primeira escolha, alinhando-se com diretrizes internacionais que recomendam sua realização dentro de 90 minutos após o primeiro contato médico.^7^ Contudo, em regiões menos desenvolvidas ou rurais, onde o acesso a centros de hemodinâmica é limitado, a fibrinólise ainda desempenha um papel crucial como estratégia de reperfusão inicial. Esta disparidade regional no acesso a recursos avançados de saúde ressalta a importância de estratégias adaptativas no manejo das SCA. A abordagem fármaco-invasiva, que combina fibrinólise inicial com transferência subsequente para ICP, emerge como uma alternativa promissora em cenários onde o acesso imediato à ICP não é viável.^8-10^

Além das questões de acesso, o estudo também destaca variações nos desfechos clínicos associados a diferentes abordagens terapêuticas. A mortalidade hospitalar e a longo prazo, bem como a incidência de eventos cardiovasculares adversos maiores (MACE), são métricas cruciais que variam não apenas com o tipo de intervenção, mas também com o tempo até o tratamento.^11^ Um aspecto particularmente relevante evidenciado pelo ACCEPT é a importância do tempo no manejo das SCA. A relação inversa entre o atraso no tratamento e os desfechos clínicos reforça a necessidade de sistemas de saúde eficientes e protocolos bem estabelecidos para o rápido diagnóstico e intervenção.^12-14^ Uma análise detalhada das taxas de reperfusão baseadas nas regiões estudadas no ACCEPT revela disparidades significativas. Enquanto regiões mais desenvolvidas, como o Sudeste e o Sul, apresentam taxas mais elevadas de ICP primária, as regiões Norte e Nordeste ainda enfrentam desafios consideráveis no acesso a esta modalidade de tratamento. Por exemplo, enquanto a taxa de ICP primária pode chegar a 70-80% em grandes centros urbanos do Sudeste, em algumas áreas do Norte e Nordeste essa taxa pode cair para 30-40% Essas disparidades nas taxas de reperfusão refletem não apenas diferenças na infraestrutura de saúde, mas também variações nos protocolos de atendimento e na capacitação dos profissionais de saúde. Além disso, é crucial considerar as comorbidades clínicas e fatores regionais que impactam a reperfusão em tempo adequado. Entre as comorbidades clínicas que frequentemente atrasam ou impedem a reperfusão adequada, destacam-se:

1. **Diabetes mellitus:** Pacientes diabéticos muitas vezes apresentam sintomas atípicos de SCA, levando a atrasos no diagnóstico e tratamento.
2. **Insuficiência renal crônica:** A presença de disfunção renal pode complicar o manejo farmacológico e aumentar o risco de complicações pós-procedimento.
3. **Idade avançada:** Pacientes idosos frequentemente apresentam manifestações atípicas da doença e têm maior risco de complicações, o que pode levar a hesitações na indicação de terapias mais agressivas.

Quanto aos fatores regionais que impactam a reperfusão adequada, podemos citar:

1. **Distância geográfica:** Em regiões remotas, o tempo de transporte até centros especializados pode exceder a janela terapêutica ideal para reperfusão
2. **Infraestrutura de saúde:** A falta de centros de hemodinâmica em algumas regiões limita o acesso à ICP primária.
3. **Capacitação profissional:** A escassez de profissionais treinados em algumas áreas pode resultar em atrasos no diagnóstico e tratamento.
4. **Fatores socioeconômicos:** Populações de baixa renda podem enfrentar barreiras adicionais no acesso aos serviços de saúde.

Em conclusão, o registro ACCEPT representa um avanço significativo na compreensão e manejo das SCA no contexto brasileiro. Seus achados devem ser utilizados para refinar protocolos clínicos, melhorar a alocação de recursos e, ultimamente, reduzir a morbimortalidade associada às doenças cardiovasculares no Brasil. A implementação de estratégias baseadas em evidências, adaptadas às realidades regionais, é fundamental para superar os desafios impostos pela vastidão territorial e diversidade socioeconômica do país. O caminho à frente exige uma abordagem multifacetada, incluindo investimentos em infraestrutura de saúde, treinamento de profissionais, educação pública sobre os sinais de alerta de SCA e o desenvolvimento de redes de atendimento integradas. Somente através de esforços coordenados e baseados em evidências, como as fornecidas pelo ACCEPT, poderemos avançar na melhoria do cuidado cardiovascular e na redução do impacto das SCA em todas as regiões do Brasil.

## References

[1] GBD 2017 Causes of Death Collaborators (2018). Global, Regional, and National Age-sex-specific Mortality for 282 Causes of Death in 195 Countries and Territories, 1980-2017: A Systematic Analysis for the Global Burden of Disease Study 2017. Lancet.

[2] Ribeiro ALP, Duncan BB, Brant LC, Lotufo PA, Mill JG, Barreto SM (2016). Cardiovascular Health in Brazil: Trends and Perspectives. Circulation.

[3] Silva PGMBE, Berwanger O, Santos ESD, Sousa ACS, Cavalcante MA, Andrade PB (2020). One Year Follow-up Assessment of Patients Included in the Brazilian Registry of Acute Coronary Syndromes (ACCEPT). Arq Bras Cardiol.

[4] Ritt LEF, Barros e Silva PGM, Darzé ES, Santos RHN, Oliveira QB, Berwanger O (2024). Myocardial Infarction with ST Elevation and Reperfusion Therapy in Brazil: Data from the ACCEPT Registry. Arq Bras Cardiol.

[5] Mattos LA (2011). Rationality and Methods of ACCEPT Registry - Brazilian Registry of Clinical Practice in Acute Coronary Syndromes of the Brazilian Society of Cardiology. Arq Bras Cardiol.

[6] Ibanez B, James S, Agewall S, Antunes MJ, Bucciarelli-Ducci C, Bueno H (2018). 2017 ESC Guidelines for the Management of Acute Myocardial Infarction in Patients Presenting with ST-Segment Elevation: The Task Force for the Management of Acute Myocardial Infarction in Patients Presenting with ST-Segment Elevation of the European Society of Cardiology (ESC). Eur Heart J.

[7] Caluza AC, Barbosa AH, Gonçalves I, Oliveira CA, Matos LN, Zeefried C (2012). ST-Elevation Myocardial Infarction Network: Systematization in 205 Cases Reduced Clinical Events in the Public Health Care System. Arq Bras Cardiol.

[8] O’Gara PT, Kushner FG, Ascheim DD, Casey DE, Chung MK, Lemos JA (2013). 2013 ACCF/AHA Guideline for the Management of ST-Elevation Myocardial Infarction: A Report of the American College of Cardiology Foundation/American Heart Association Task Force on Practice Guidelines. Circulation.

[9] Bianco HT, Povoa R, Izar MC, Alves CMR, Barbosa AHP, Bombig MTN (2022). Pharmaco-invasive Strategy in Myocardial Infarction: Descriptive Analysis, Presentation of Ischemic Symptoms and Mortality Predictors. Arq Bras Cardiol.

[10] Byrne RA, Rossello X, Coughlan JJ, Barbato E, Berry C, Chieffo A (2023). 2023 ESC Guidelines for the Management of Acute Coronary Syndromes. Eur Heart J.

[11] Jneid H, Addison D, Bhatt DL, Fonarow GC, Gokak S, Grady KL (2017). 2017 AHA/ACC Clinical Performance and Quality Measures for Adults with ST-Elevation and Non-ST-Elevation Myocardial Infarction: A Report of the American College of Cardiology/American Heart Association Task Force on Performance Measures. J Am Coll Cardiol.

[12] Ting HH, Bradley EH, Wang Y, Lichtman JH, Nallamothu BK, Sullivan MD (2008). Factors Associated with Longer Time from Symptom Onset to Hospital Presentation for Patients with ST-Elevation Myocardial Infarction. Arch Intern Med.

[13] Pinto DS, Kirtane AJ, Nallamothu BK, Murphy SA, Cohen DJ, Laham RJ (2006). Hospital Delays in Reperfusion for ST-Elevation Myocardial Infarction: Implications When Selecting a Reperfusion Strategy. Circulation.

[14] Armstrong PW, Gershlick AH, Goldstein P, Wilcox R, Danays T, Lambert Y (2013). Fibrinolysis or Primary PCI in ST-Segment Elevation Myocardial Infarction. N Engl J Med.

