# Supplementary material for: O Estudo ACCEPT e o Manejo da Doença Isquêmica Miocárdica no Brasil: Desafios e Oportunidades em um País Continental
Source: Arq Bras Cardiol. 2024 Nov 13;121(11):e20240604. [Article in Portuguese] doi: 10.36660/abc.20240604 (PMC11634297; doi:10.36660/abc.20240604)
Supplement: Translation

# Translation: The ACCEPT Study and the Management of Ischemic Myocardial Disease in Brazil: Challenges and Opportunities in a Continental Country

**DOI:** 10.36660/abc.20240604i

**Authors:** Henrique Tria Bianco

**Affiliations:** 1 São Paulo SP Brazil Escola Paulista de Medicina – Universidade Federal de São Paulo, São Paulo, SP – Brazil

**Keywords:** Myocardial Infarction, Acute Coronary Syndrome, Medical Record Linkage

Ischemic myocardial disease, particularly in its acute manifestation as an acute coronary syndrome (ACS), remains one of the main public health challenges in Brazil and the world.^1,2^ In this context, the ACCEPT (Acute Coronary Syndrome Registry) study emerges as a crucial source of epidemiological and clinical information on the management of ACS in the Brazilian scenario, with the aim of evaluating evidence-based therapies, the occurrence of outcomes, use of reperfusion and predictors for not receiving reperfusion in patients with ACS in a multicenter national registry.^3,4^

Brazil, with its continental dimensions and marked regional disparities, presents a unique challenge in implementing uniform strategies for the treatment of SCA. The study ACCEPT not only provides valuable data on the prevalence and types of ACS found in the country but also sheds light on the different therapeutic approaches adopted in different regions.^5^ Myocardial revascularization is a fundamental pillar in the treatment of ACS, especially in ST-segment elevation myocardial infarction (STEMI).^6^ ACCEPT demonstrates that, although primary pPCI (Primary Percutaneous Coronary Intervention) is considered the gold standard for STEMI, its availability and application vary significantly between Brazilian regions. In metropolitan areas and referral centers, primary PCI is often the first choice, in line with international guidelines that recommend its performance within 90 minutes after first medical contact.^7^ However, in less developed or rural regions, where access to hemodynamic centers is limited, fibrinolysis still plays a crucial role as an initial reperfusion strategy. This regional disparity in access to advanced healthcare resources highlights the importance of adaptive strategies in the management of ACS. The pharmaco-invasive approach, which combines initial fibrinolysis with subsequent transfer for PCI, emerges as a promising alternative in settings where immediate access to PCI is not feasible.^8-10^

In addition to access issues, the study also highlights variations in clinical outcomes associated with different therapeutic approaches. In-hospital and long-term mortality, as well as the incidence of major adverse cardiovascular events (MACE), are crucial metrics that vary not only with the type of intervention but also with time totreatment.^11^ A particularly relevant aspect highlighted by ACCEPT is the importance of time in the management of ACS. The inverse relationship between treatment delay and clinical outcomes reinforces the need for efficient health systems and well-established protocols for rapid diagnosis and intervention.^12-14^ A detailed analysis of reperfusion rates based on the regions studied in ACCEPT reveals significant disparities. While more developed regions, such as the Southeast and South, have higher rates of primary PCI, the North and Northeast regions still face considerable challenges in accessing this modality of treatment. For example, while the rate of primary PCI can reach 70-80% in large urban centers in the Southeast, in some areas of the North and Northeast, this rate can drop to 30-40%. These disparities in reperfusion rates reflect not only differences in health infrastructure but also variations in care protocols and the training of health professionals. In addition, it is crucial to consider clinical comorbidities and regional factors that impact timely reperfusion. Among the clinical comorbidities that frequently delay or prevent adequate reperfusion, the following stand out:

1. **Diabetes mellitus:** Diabetic patients often present with atypical symptoms of ACS, leading to delays in diagnosis and treatment.
2. **Chronic renal failure:** The presence of renal dysfunction may complicate pharmacological management and increase the risk of post-procedure complications.
3. **Advanced age:** Elderly patients often present with atypical manifestations of the disease and are at greater risk of complications, which may lead to hesitation in prescribing more aggressive therapies

Regarding regional factors that impact adequate reperfusion, we can mention:

1. **Geographical distance:** In remote regions, transportation time to specialized centers may exceed the ideal therapeutic window for reperfusion
2. **Health infrastructure:** The lack of hemodynamic centers in some regions limits access to primary PCI.
3. **Professional training:** Shortages of trained professionals in some areas can result in delays in diagnosis and treatment.
4. **Socioeconomic factors:** Low-income populations may face additional barriers to accessing health services.

In conclusion, the ACCEPT registry represents a significant advance in the understanding and management of ACS in the Brazilian context. Its findings should be used to refine clinical protocols, improve resource allocation, and ultimately reduce morbidity and mortality associated with cardiovascular diseases in Brazil. Implementing evidence-based strategies adapted to regional realities is essential to overcome the challenges posed by the country's vast territorial size and socioeconomic diversity. The path forward requires a multifaceted approach, including investments in health infrastructure, training of professionals, public education on the warning signs of ACS, and the development of integrated care networks. Only through coordinated and evidence-based efforts, such as those provided by ACCEPT, will we be able to advance in improving cardiovascular care and reducing the burden of ACS in all regions of Brazil.
